# Consensus statement on the epidemiology, diagnosis, prevention, and management of cow's milk protein allergy in the Middle East: a modified Delphi-based study

**DOI:** 10.1007/s12519-021-00476-3

**Published:** 2021-11-24

**Authors:** Moustafa A. El-Hodhod, Mortada H. F. El-Shabrawi, Ahmed AlBadi, Ahmed Hussein, Ali Almehaidib, Basil Nasrallah, Ebtsam Mohammed AlBassam, Hala El Feghali, Hasan M. Isa, Khaled Al Saraf, Maroun Sokhn, Mehdi Adeli, Najwa Mohammed Mousa Al-Sawi, Pierre Hage, Suleiman Al-Hammadi

**Affiliations:** 1 Department of Pediatrics, Faculty of Medicine, Ain Shams University, Cairo, Egypt; 2 Faculty of Medicine, October 6 University, Giza, Egypt; 3 Faculty of Medicine, Cairo University, Cairo, Egypt; 4 International Pediatric Association (IPA), Marengo, USA; 5 International Society of Tropical Pediatrics (ISTP), Manila, Philippines; 6 DRC Hospital, Muscat, Oman; 7 Al Amiri and Al Adan Hospitals, Kuwait, Kuwait; 8 Pediatric Gastroenterology, Department of Pediatrics, King Faisal Specialist Hospital and Research Center Riyadh, Riyadh, Saudi Arabia; 9 American Hospital Dubai, Dubai, United Arab Emirates; 10 Nutrition Service Department, King Faisal Specialist and Research Center Riyadh, Riyadh, Saudi Arabia; 11 CHU Notre Dame de Secours, Byblos, Lebanon; 12 Pediatric Department, Salmaniya Medical Complex, Manama, Bahrain; 13 Pediatric department, Arabian Gulf University, Manama, Bahrain; 14 Pediatrics Department, Dar Al Shifa Hospital, Kuwait, Kuwait; 15 Pediatric Gastroenterology, Hepatology, and Nutrition Department, Saint George University Medical Center, University of Balamand, Beirut, Lebanon; 16 Sidra Medicine, Doha, Qatar; 17 Department of Pediatrics, Doctor Sulaiman Alhabib Medical Group, Alrryan Branch, Riyadh, Saudi Arabia; 18 Pediatric Pulmonology and Allergology, University of Balamand, Beirut, Lebanon; 19 College of Medicine, Mohammed Bin Rashid University of Medicine and Health Sciences, Building 14, 505055 Dubai, United Arab Emirates

**Keywords:** Consensus Cow’s milk protein allergy, Infant formula, Middle East, Milk hypersensitivity

## Abstract

**Background:**

This study aimed to develop an expert consensus regarding the epidemiology, diagnosis, and management of cow’s milk protein allergy (CMPA) in the Middle East.

**Methods:**

A three-step modified Delphi method was utilized to develop the consensus. Fifteen specialized pediatricians participated in the development of this consensus. Each statement was considered a consensus if it achieved an agreement level of ≥ 80%.

**Results:**

The experts agreed that the double-blind placebo-controlled oral challenge test (OCT) should be performed for 2–4 weeks using an amino acid formula (AAF) in formula-fed infants or children with suspected CMPA. Formula-fed infants with confirmed CMPA should be offered a therapeutic formula. The panel stated that an extensively hydrolyzed formula (eHF) is indicated in the absence of red flag signs. At the same time, the AAF is offered for infants with red flag signs, such as severe anaphylactic reactions. The panel agreed that infants on an eHF with resolved symptoms within 2–4 weeks should continue the eHF with particular attention to the growth and nutritional status. On the other hand, an AAF should be considered for infants with persistent symptoms; the AAF should be continued if the symptoms resolve within 2–4 weeks, with particular attention to the growth and nutritional status. In cases with no symptomatic improvements after the introduction of an AAF, other measures should be followed. The panel developed a management algorithm, which achieved an agreement level of 90.9%.

**Conclusion:**

This consensus document combined the best available evidence and clinical experience to optimize the management of CMPA in the Middle East.

## Introduction

Cow's milk protein allergy (CMPA) is an abnormal immunological response to specific proteins, mainly casein and/or whey proteins, present in either formula or breast milk [1]. The current epidemiological figures highlight that CMPA is the prevalent form of food hypersensitivity in children younger than three years, affecting up to 7.5% of them in the first year of life [2]. In some Middle Eastern countries, the incidence of CMPA among infants younger than 2 years was reported to be 3.4% [3]. Positive family history of atopy and atopic dermatitis in early infancy are distinguished risk factors for CMPA [4, 5]. Based on the type of immunological reactions, the clinical presentation of the CMPA can be broadly divided into immediate and delayed-onset presentations. Eczema and allergic colitis are commonly present in breastfed infants [6].

CMPA is a clinical condition in which proper history taking and physical examination are the cornerstones for accurate identification of the patients [7]. However, the diagnosis of CMPA can be challenging, and further investigations are usually requested [2, 7, 8]. The management of CMPA is usually tailored according to the type of feeding and age of affected patients [8, 9]. Correct identification and management of CMPA are crucial to optimize infant growth and to prevent severe complications.

In the Middle East, it was reported that the practice of exclusive breastfeeding up to 6 months of life is poorly followed [10]. The early introduction of cow's milk can significantly increase the risk of CMPA among infants from the Middle East; in addition, the utilization of other forms of milk, such as goat's milk, is rather common in this region, which may exhibit cross-reactivity with cow's milk [11, 12]. Thus, it is imperative to develop a consensus to aid general practitioners and pediatricians in the diagnosis and management of CMPA in the Middle East region. Although previous consensus documents for the diagnosis and management of CMPA from the Middle East region were published [10, 13], they did not utilize the Delphi-based approach, which offers the advantages of systematic approach and subject anonymity during voting [14].

Thus, we conducted a Delphi method-based study to develop a consensus regarding the epidemiology, diagnosis, and management of CMPA in the Middle East. A three-step Delphi survey was adopted to integrate the opinions of experts and to formulate a clinical pathway algorithm for diagnosis and management of CMPA that presents to primary and advanced healthcare settings in the Middle East. Moreover, we aimed to record the unmet medical needs concerning CMPA management.

## Methods

### Study design

A three-step modified Delphi method was utilized to develop the present consensus through the period from September to December 2020. This study consisted of two rounds of an anonymous voting and a virtual expert discussion meeting to develop the consensus statements and the clinical pathway algorithm.

### Expert panel recruitment

A non-probability purposive sampling technique was conducted to recruit 15 specialized pediatricians from the following countries: Egypt, Kingdom of Saudi Arabia, United Arab Emirates (UAE), Lebanon, Qatar, Bahrain, and Kuwait. All experts were required to have an active research profile in the field of pediatric immunology and gastroenterology, and to be affiliated with an academic institution from the Middle East region. Eligible experts were invited via email to participate and were asked to participate in the three steps of the Delphi method-based study.

### Survey development

A systematic literature search was employed on Medline via PubMed from its inception to September 2020 to collect relevant information by the survey development committee. Various combinations of the following keywords were used to identify potentially eligible literature: ("Cow's milk protein allergy" OR "allergy, milk[MeSH Terms]" OR "(allergies, milk[MeSH Terms])") and ("epidemiology" OR "incidence" OR "features" OR "diagnosis" OR "diagnostic tests" OR "Skin prick test" OR "Serum Ig-E" OR "Food challenge" OR "elimination diet" OR "prevention" OR "Management" OR "Extensively hydrolyzed formula" OR "Amino acid–based formula" OR "Formula"). The statements were primarily extracted from studies with level 1 quality of evidence, as classified by Wright et al.[15]. Additional statements were retrieved from studies with a lower quality of evidence whenever deemed required by the survey development committee. All statements were collected in an Excel spreadsheet, and the committee held a meeting to finalize the draft consensus statements.

### Voting rounds

The development of the consensus document passed through three steps. In the first step, a draft questionnaire was sent to experts via email. The questionnaire consisted of binary statements for which the experts were asked to choose between “agree” and “disagree” options. Each expert was able to comment on each statement and to provide suggestions. Each statement was considered a consensus if it achieved an agreement level of ≥80% [16]. The statements that did not achieve the agreement level were persevered for step 2 to be modified or omitted by the experts. A virtual advisory board meeting was conducted in the second step and engaged all experts on the 30th of October 2020. The meeting was divided into two parts. In the first part, the statements that achieved ≥80% agreement were presented for full consensus by the panel, while the remaining statements were presented for modification or omission. The second part of the meeting aimed to develop the clinical pathway algorithm for patients presenting with CMPA. In the final step, the list of modified statements and the clinical pathway algorithm were emailed to the experts for voting and followed the same voting process of step 1.

## Results and discussion

### Epidemiology

CMPA is the most prevalent food allergy (FA) of young children [17]. In infants less than one year of age, two cohort studies showed that the prevalence of CMPA ranged between 2.2 and 2.8%, which is consistent with the findings of another cohort of approximately 6000 newborns followed for 34 months [18, 19]. Many systematic reviews and meta-analyses were conducted to assess the prevalence of CMPA globally. Rona et al. conducted a pooling analysis of 51 studies to assess the worldwide prevalence of FA [17]. Their findings showed that the prevalence of self-reported CMPA ranged between 1.2 and 17%. This estimate was significantly lower in the studies that reported their prevalence based on symptomatic evaluation (0–2%), food challenge (0–3%), and skin prick test (SPT) sensitization and IgE assessment (2–9%). In the meta-analysis of Nwaru et al. [20], the overall effect estimate of 42 primary articles on CMPA demonstrated that the prevalence of self-reported CMPA was 2.3% (95% CI 2.1–2.5), food challenge was 0.6% (95% CI 0.5–0.8), SPT alone was 0.3% (95% CI 0.03–0.6), and by sIgE alone, it was 4.7% (95% CI 4.2–5.1). A higher prevalence has been shown among younger ages. Both studies concluded that the observed variation is attributed to the varying factors including the study design, source of population, age of participants, geographical region, and diagnosis limitations.

In the Middle East, Katz et al. conducted a single-center prospective study, which identified that the cumulative incidence of CMPA over 2 years of follow-up was 0.5% in Israeli infants. They also showed that the mean age of onset was 4 months [21]. In Oman, sensitization to cow milk was reported in 78/164 patients. This prevalence may be overestimated owing to the small sample size and the diagnostic test used [22]. In Kuwait, a survey of self-reported FA showed that out of 865 participants, 104 reported FA. Of them, 46.7% had CMPA. The prevalence in early childhood was 21.9%, and in late childhood, it was 20.8% [23]. In Lebanon, the prevalence of self-reported CMPA was 14%. It ranked as the fourth common allergen [24]. Zeyrek et al., reported that the prevalence of CMPA was 0.16% in children under 2 years of age in Turkey [25].

The experts stated that the estimated prevalence of CMPA in the Middle East ranges from 1 to 5% (level of agreement = 86.7%, Table 1).

**Table 1:** Middle East Consensus Statements on epidemiology and diagnosis of Cow's milk protein allergy (CMPA)

Statement	Level of agreement (%)
**Epidemiology**	
The prevalence of CMPA in the Middle East ranges from 1% to 5%	86.70
**Clinical presentation of CMPA**	
Physicians should suspect an increased risk for CMPA if there is a positive family history of atopy, especially in first-degree relatives	100.00
The onset of symptoms of IgE-mediated reactions could rapidly evolve within minutes to hours after cow milk protein ingestion. On the other hand, the onset of symptoms in non-IgE-mediated CMPA could be delayed for days or weeks. However, there is an apparent delay in the proper and early diagnosis of CMPA, which could reach up to 6 months	100.00
CMPA should be suspected if: (1) Symptoms developing after the introduction of cow’s milk; (2) More than one organ system is involved; (3) Symptoms are not responding to specific treatment in monosymptomatic infants, and (4) Family history of atopy	90.90
In some infants, irritability, colic, and GERD may be the only symptoms of food allergy after excluding other causes	90.90
Skin and gastrointestinal manifestations commonly exist in infants with CMPA occurring in up to 50% of patients. However, respiratory manifestations are less common in infants with CMPA occurring in less than 25% of patients	100.00
Diagnosis of CMPA should be based on symptoms. However, several available tests could add value when diagnosing CMPA	100.00
**Investigations**	
***Serum IgE and SPT***	
The determination of specific IgE in a blood sample and the SPT are useful diagnostic tests. However, both tests are less reliable in patients younger than 6 months of age	87.50
Skin prick test and specific IgE titers are helpful in predicting the prognosis and the time interval until the next oral food challenge	100.00
Neither the determination of total IgE nor the ratio of specific IgE to total IgE offers a benefit over specific IgE alone in the diagnostic workup of CMPA	100.00
In highly atopic infants, the confirmatory cow’s milk protein challenge can be postponed until the child shows a reduced reaction in the tests for cow’s milk protein-specific IgE	85.70
Intradermal testing should not be performed in highly sensitized individuals because it carries a risk of a systemic allergic reaction	84.60
***Endoscopy and biopsy***	
Upper and/or lower endoscopies with multiple biopsies are indicated in patients with: (1) Unexplained significant and persistent gastrointestinal symptoms, (2) Significant failure to thrive not improving with treatment (3) Significant iron-deficiency anemia not responding to adequate iron therapy	90.90
***Oral challenge test***	
The starting dose during an oral milk challenge in children with a delayed reaction should be increased stepwise to 100 mL (e.g., stepwise doses of 1, 3, 10, 30, and 100 mL given at 30-minute intervals)	93.30
If severe reactions are expected, then the challenge should begin with minimal volumes (e.g., stepwise dosing of 0.1, 0.3, 1.0, 3.0, 10.0, 30.0, and 100 mL given at 30-minute intervals)	100.00
Challenges should be preferably carried out in a hospital in the following circumstances: (1) A history of immediate allergic reactions, (2) Unpredictable reaction, (3) Severe atopic eczema	86.70
In a case of previous anaphylaxis, the challenge is contraindicated unless SPTs and/or specific IgE measurements showed improvement	85.70
A child should be given a strict CMP-free diet for a period of at least 6 months–1 year before an oral food challenge is performed	93.30
***Diagnostic elimination***	
The elimination diet should be continued for a minimum of at least 2 weeks and up to 4 weeks in cases of atopic dermatitis or allergic colitis, respectively	100.00
In formula-fed CMPA infants, cow’s milk-based formula and supplementary foods containing cow’s milk protein or other unmodified animal milk proteins (e.g., goat’s milk, sheep’s milk) should be strictly avoided	100.00
An eHF may be considered as the first choice in most cases with CMPA, predominantly because it is less expensive than the AAF and has shown efficacy at inducing tolerance	86.70
An AAF is indicated if: (1) the child refused the taste of the eHF and accepted the AAF; (2) the symptoms did not improve on the eHF after 2–4 weeks, and (3) the cost–benefit ratio favors the AAF over the eHF	100.00
If there is no improvement with eHF for 2–4 weeks, then an allergic reaction to the peptides must be considered; and AAF should be tried before CMPA is ruled out as a cause of the symptoms	100.00
The response to the introduction of an AAF enables the formula to be considered as a diagnostic tool for CMPA. If the symptoms did not disappear on the AAF, the diagnosis of CMPA should be revised	100.00
Effective treatment can be established over a reduced period using a diagnostic elimination with an AAF; it reduces cost and shortens the duration of symptoms	92.30
If the child refused the AAF, several trials of feeding should be tried, including a suitable flavoring agent with gradually increasing the volume of milk till acceptance. In case of the complete refusal of a certain formula, a trial of another AAF formula should be considered to ensure sufficient intake	100.00
In exclusively breastfed infants, mothers should be encouraged to continue breastfeeding while avoiding all dairy (milk) and milk products from their own diet during the diagnostic elimination diet	100.00
In extremely sick, exclusively breastfed infants, AAF is recommended for diagnostic elimination to stabilize the infant’s condition during the period when the breastfeeding mother is transitioning to a cow’s milk protein-free diet	92.30
Continuing breastfeeding should always be encouraged. However, in rare cases, if breastfed infants with severe symptoms (e.g., severe atopic eczema or allergic (entero) colitis complicated by growth faltering and/or hypoproteinemia and/or severe anemia) did not improve after maternal diet elimination, a trial of AAF is recommended for a period of several days to a maximum of 2 weeks	100.00
In certain rare occasions, exclusively breastfed infants may present with severe symptoms complicated by growth faltering, hypoproteinemia, and/or severe anemia. Patients with severe symptoms should be referred to a specialist to exclude other pathologies before suspecting CMPA	90.90
In case of availability, soy protein-based formula is an option in infants older than 6 months who do not accept the taste of an eHF. However, extreme caution should be taken due to the high cross-reactivity of soy protein-based formulas with CMP	80.00
In children older than 2 years with persistent CMPA, an elimination diet can be provided by solid foods and liquids free of CMP unless the child has multiple allergies	100.00
In children older than 2 years, if multiple food allergies are suspected, exclusive feeding with an AAF should be considered to allow symptoms improvement before an oral challenge with allergens	92.90

*CMPA* cow’s milk protein allergy, *GERD* gastrointestinal esophageal reflux, *SPT* skin prick test, *AAF* amino acid formula, *eHF* extensively hydrolyzed formula

### Diagnosis

For CMPA, there is no single test or biomarker that is pathognomonic of the condition [8]. The cornerstones of the CMPA diagnosis are the reliable history and the proper physical examination [26]. A systematic approach is required for an accurate diagnosis and should begin with an allergy-focused history and physical examination. Many differential diagnoses should be considered, including immune deficiency, gastroesophageal reflux disease, infective colitis, and eosinophilic esophagitis [27]. In the case of non-definitive diagnosis, empirical exclusion therapy is not an evidence-based practice and should be avoided.

#### Clinical presentations of CMPA

Clinical features of CMPA are usually present within a few days or months of life after the introduction of a cow's milk-based formula. Moreover, the same symptoms can occur if the cow’s milk protein (CMP) is transmitted from the maternal diet to the infant through maternal breast milk [28]. Several IgE- and non-IgE-mediated clinical syndromes are found to be associated with CMPA patients. In patients with IgE-mediated, the most common clinical presentations are urticaria, anaphylaxis, angioedema, oropharyngeal or gastrointestinal reactions, and food-associated anaphylaxis [29]. In patients with non-IgE-mediated CMPA, gastrointestinal reflux, colic, constipation, food protein-induced enteropathy are frequent [30]. Atopic dermatitis and eosinophilic gastrointestinal disorders are seen in patients with mixed IgE and non-IgE-mediated CMPA [31].

#### Investigations

##### Oral food challenge (OFC)

Diagnostic approaches of CMPA are limited and affect the ability to explain the underlying epidemiology. A double-blind, placebo-controlled oral food challenge (DBPCFC) is the gold standard and the most specific test for diagnosis [26]. In young children, open OFC is well-validated with some concerns. Clinical allergy and sensitization can be differentiated using OFC [32]. Nevertheless, DBPCFC and OFC require a longer time to perform and should be done under medical observation as they are associated with a risk of anaphylaxis [33]. Therefore, the OFCs are not always ideal for clinical practice. A therapeutic elimination diet should be applied immediately in cases of severe anaphylaxis. After the elimination period, the test should be repeated to confirm the tolerance development [34]. Using OFC, CMPA can be ruled out if the patient remains without symptoms for 2 weeks [35]. Nevertheless, the diagnosis of CMPA is proven if the symptoms arise.

##### Serum-specific IgE and skin prick test (SPT)

Studies using these approaches may have inadequate evaluations of extremely atopic children owing to parental refusal and safety issues. In both epidemiological and therapeutic trials, targets include serum-specific IgE and SPT [27]. The presence of IgE tissue-bound antibodies and circulating antibodies is detected by SPT and specific IgE (sIgE), respectively [36]. These two measures estimate the probability of reaction but are not diagnostically sufficient alone. The SPT-measured sensitization is also described as at least 3 mm wheel larger than the negative control [37]. IgE binding to specific proteins is calculated by cow's milk-specific IgE, measured by in vitro immunoassay; sensitization is characterized as measurable specific IgE (often sIgE is ≥ 0.35 kU/L, sometimes ≥ 0.10 kU/L) [38]. However, positive IgE neither confirms any allergy nor distinguishes sensitization from clinical allergy [8, 28].

In the diagnosis of non-IgE-mediated CMPA, specific IgE tests are not useful [39]. In terms of SPT, a positive test does not confirm an allergy, especially in infants. Moreover, in non-IgE-mediated CMPA, SPT may lead to false-positive or false-negative diagnosis [40]. A definitive diagnosis of IgE-mediated CMPA is confirmed by the history of an instant reaction with classic allergic symptoms and positive sIgE or SPT tests [41]. CMPA self-reporting and sensitization based only on serum IgE and SPT to detect CMPA seems to overestimate the prevalence [17]. Furthermore, differences in the sIgE tests may result in conflicting interpretations and may restrict comparability.

#### Endoscopy and biopsy

In infants, CMPA can result in occult blood in stools, which can be detected until 6–12 weeks after avoidance of CMP. After 3 weeks, sigmoidoscopy is indicated if the occult blood is still detected [2]. Other indications for upper and lower endoscopy include persistent malabsorption or malnutrition, nutrient deficiency, inadequate weight gain, malnutrition, persistent anemia, suspected eosinophilic gastroenteropathy or eosinophilic esophagitis, persistent abdominal pain and bloating, hyporexia, and suspected inflammatory bowel disease [42]. CMPA cases may exhibit signs suggestive of eosinophilic esophagitis in the endoscopic examination, such as circular rings and altered vascular patterns. In patients with initial presentation of persistent vomiting, upper endoscopy may be suggested to exclude surgical cases or eosinophilic gastroenteritis. In patients with CMPA accompanied with gastrointestinal manifestations, both sigmoidoscopy and rectal biopsy were reported to be predictive [43]. In the majority of the patients, the histological examination may reveal focal erythema or nodular lymphoid hyperplasia [44]. CMPA is confirmed in the existence of more than 15–20 eosinophils per high power field or more than 60 eosinophils in six high power fields [27].

### Scoring system for screening

There are many scoring systems for the CMPA; however, Cow’s milk-related symptom score (CoMiSS), a simple, fast, and easy-to-use awareness tool, is the most common [26]. However, until now, there is no consensus on the cut-off values for this tool. Besides this, the sensitivity and specificity of the CoMiSS tool are poor. Therefore, it cannot be recommended as a screening tool until more studies are available.

### Diagnostic elimination

In exclusively breastfed infants, CMPA is typically mild and not associated with failure to thrive or anemia [45]. It is recommended to advise the mother to avoid consumption of dairy products and/or bovine milk in the first 6 months. Moreover, mothers should avoid dairy or milk-containing foods in their diet [46]. In response to the elimination of milk and its products, the antigens may disappear within 72 hours from the mother’s breast milk. Following the elimination, if the symptoms improved, it is recommended to re-introduce the suspected allergen to the mother’s diet in the case of a breastfed infant or to re-introduce the previously used formula [41]. The recurrence of symptoms can confirm the diagnosis of CMPA and should be followed by eliminating the allergen and continuous breastfeeding. However, if the previous reaction is severe or life-threatening, this circulation should be omitted until the reaction is consistent or the patient is transferred to an experienced center. The elimination diet should be maintained for at least six months or until 12 months of age [47]. However, in patients with IgE-mediated CMPA, an elimination diet should be maintained for 18 months. In formula-fed cases, the recommended formulas are extensively hydrolyzed formula (eHF), or an AAF in case of severe symptoms [26]. During the diagnostic elimination period, cow’s-milk and cow’s-milk-containing formula and supplementary foods should be strictly forbidden in non-breastfed infants [48]. If the first feed in a formula-fed baby causes signs of CMPA, the formula should be modified, and the elimination should be made in the infant's diet, not in the mother's diet [26].

Many hidden allergens can cause CMPA; therefore, the counseling of an experienced, clinically trained pediatric dietitian is strongly recommended. For children over two years with persistent severe CMPA, solid foods and liquids free of cow’s milk protein should be given unless the child has multiple allergies [8]. Besides these interventions, goat's and sheep's-milk protein should be strictly avoided due to the high cross-reactivity with cow’s milk protein [49]. In cases of highly atopic children or children with eosinophilic digestive tract disorders, if multiple FA is suspected, an exclusive feeding with an AAF may be considered to improve the symptoms before an oral challenge with cow’s milk is performed [48, 50].

The statements regarding the epidemiology and diagnosis of CMPA that achieved a consensus agreement are provided in Table 1. The panel emphasized the importance of early diagnosis and exclusive or partial breastfeeding. The panel agreed that infants with a positive family history of atopy in first-degree relatives are at increased risk of CMPA. Despite the significant impact of early diagnosis on the outcome of CMPA, the experts stated there is an apparent delay in the diagnosis of CMPA in the Middle East, which can be extended up to 6 months. Infants with positive family history, who exhibit allergic symptoms after the introduction of cow’s milk involving at least two systems and not responding to treatment, should be suspected for CMPA. Dermatological and gastrointestinal manifestations commonly present in CMPA, while respiratory manifestations occur in less than one-fourth of the patients. However, general pediatricians and primary healthcare physicians should be aware that unexplained gastrointestinal symptoms may be the only presentation of FA.

Although the experts stated that the CMPA is a clinical diagnosis, they highlighted that a number of available tests could add diagnostic and prognostic values, including specific IgE levels and SPT in infants older than 6 months. However, in highly atopic infants, intradermal testing should not be performed. The existence of persistent or severe unexplained gastrointestinal symptoms is an indication for endoscopy with biopsies if it co-exists with failure to thrive or refractory iron-deficiency anemia.

The diagnostic elimination diet should be continued for 2–4 weeks and should be deprived of CMP or other unmodified animal milk proteins; nonetheless, the confirmatory CMP challenge can be postponed in highly sensitized infants until the child shows reduced levels of specific IgE. In case of persistent or severe symptoms, an AAF should be tried for a maximum of 2 weeks before CMPA is ruled out. Additional indications for an AAF are when the child refuses the taste of the eHF or when the cost–benefit ratio favors the use of an AAF.

During an elimination diet, mothers of exclusively breastfed infants should continue breastfeeding while maintaining a restricted diet. However, in rare cases, if breastfed infants with severe symptoms did not improve after maternal diet elimination, a trial of AAF is recommended for a period of several days to a maximum of 2 weeks.

In addition, the experts agreed that the starting dose during an oral milk challenge in children with a delayed reaction should be increased stepwise to 100 mL. If severe reactions are expected, the challenge should begin with minimal volumes.

### Management of at-risk infants and the role of hydrolyzed formulas

Untreated CMPA can increase the risk of allergic disorders later in life [51]. The timing of the development of CMPA is a major determinant of growth retardation, with earlier CMPA development carrying a greater risk of growth retardation [7]. CMPA also can adversely affect the quality of life of infants and their families [52]. Thus, the identification and monitoring of at-risk patients are crucial to optimize the outcomes of CMPA. Several risk factors are incorporated in the development of CMPA, including a positive family history of atopy, prematurity, multi-parity, advanced maternal age, mother's education, formula feeding, and short time of exclusive breastfeeding [10, 53].

Exclusive breastfeeding for 4–6 months is a widely recommended, cost-effective, strategy for at-risk infants. Previous reports showed that exclusive breastfeeding was associated with a significant reduction in the risk of cow's milk sensitization and atopic dermatitis [54–56]. Nonetheless, the current body of evidence still shows conflicting results regarding the beneficial role of exclusive breastfeeding in preventing FA [57, 58]. According to the recent European Academy of Allergy and Clinical Immunology (EAACI), there is no recommendation for or against using breastfeeding to prevent FA; nonetheless, exclusive breastfeeding should be universally recommended owing to its outstanding benefits for both mother and infant [59].

On the other hand, modification of maternal diet is not recommended as a general preventive strategy for FA [29]; the current body of evidence demonstrates a negative impact of diet restriction on maternal and fetal weight [60]. Nonetheless, previous systematic reviews have highlighted that an antigen avoidance diet can reduce the risk of allergic diseases in at-risk infants; however, these findings should be interpreted cautiously owing to the low quality of supporting evidence [60]. Nonetheless, a recent review concluded that avoiding potential food allergens may have little to no effect on the risk of CMPA [59].

In formula-fed infants with a high risk of CMPA, usually defined as a positive family history of atopy among first-degree relatives, it was previously thought that CMP should be avoided in the diets (by using hydrolyzed formula) to prevent CMPA development [10]. According to recent EAACI guideline, the introducing CMP-based formula after the first week of life did not have a consistent impact on the development of CMPA in infancy or early childhood, with no significant harms after three months of age [59].

Hydrolyzed formulas are manufactured by variable degrees of hydrolysis of milk protein to smaller peptides. By exposing the gut-associated lymphoid tissue (GALT) to such small peptides, hydrolyzed formulas can induce oral tolerance without sensitization, with a subsequent decrease in the risk of atopic diseases [61]. According to the degree of hydrolysis, these formulas are classified into partially hydrolyzed formulas (pHF; peptides size < 5 kDa) or eHF (peptides size < 3 kDa) [62]. The eHF is characterized by minimum allergenicity and, hence, is more preferred during the treatment of CMPA. On the other hand, pHF is theoretically associated with more immunogenicity and can lead to greater induction of oral tolerance than eHF [63, 64]. In addition, pHF is thought to be easily digested and has the advantages of better gastrointestinal tolerance than cow's milk formula (CMF) or other standard formulas [65]. Several randomized controlled trials showed no significant difference in atopic dermatitis incidence between different hydrolyzed formulas or between hydrolyzed formulas and CMF [61, 66, 67]. According to the recent EAACI guideline, pHF or eHF, whey or casein, may not reduce the risk of CMPA compared with conventional CMF [59]. Therefore, no recommendation exists for or against using pHF or eHF to prevent CMPA in infants. When exclusive breastfeeding is not possible, many substitutes are available to utilize, including hydrolyzed formulas. The EAACI also recommended that supplementation with regular cow's milk formula may be avoided in the first week of life.

In a small set of infants, hydrolyzed formulas can trigger immunological reactions due to residual proteins. In these conditions, the AFF has been proposed as an alternative formula to avoid hypersensitivity [68, 69]. However, there are no data to support its use for preventing allergic diseases in at-risk infants [13].

The current body of evidence demonstrated substantial heterogeneity in the benefits of diet restriction and timing of introduction of solid food on the outcomes of CMPA among at-risk infants [70–72]. Delayed introduction of the food can lead to nutritional imbalance and growth deficits [29]. Thus, the previous consensus from the Middle East did not recommend delayed introduction of solid food beyond the first 4–6 months of life [13].

The intestinal microbiome is an integral part of the development process during the first year of life. Previous studies have established a strong association between microbiome dysfunction and the development of allergic diseases [73, 74]. Thus, a growing number of literature evaluated the role of prebiotics-rich formulas and probiotic supplements during pregnancy on the incidence of FA among at-risk infants [75, 76]. Hypothetically, prebiotics can selectively induce the growth of a beneficial gut microbiome, with a subsequent reduction in the risk of eczema and other allergic diseases [73]. Concerning the availability of clinical evidence, previous consensuses highlighted that no data exist to support the use or avoidance of prebiotic supplements during pregnancy and lactation [77]. In exclusively breastfed infants, the prebiotics did not exhibit clear benefits concerning the prevention of allergic diseases [78].

### Management of CMPA

Early diagnosis is a major determinant of the course of CMPA. A delay in the diagnosis of CMPA significantly increases the risk of growth retardation, anemia, and hypoproteinemia [79]. In both formula and breastfed infants, strict avoidance of a CMP-containing diet is the cornerstone for the management of CMPA [80]. The management is then tailored according to the age of the patients, type of feeding, type of hypersensitivity reactions, and the severity of clinical symptoms [69].

It is universally recommended to breastfeed infants with CMPA [13]. In exclusively breastfed infants, mothers are instructed to avoid any CMP-containing diet, such as cow’s milk products including cheese and yogurt, due to the excretion of cow's milk peptides in breast milk [81]. If the CMP-containing diet is prolonged, it should be accompanied by nutritional counseling to assess the mother's needs for dietary supplements to replenish micronutrients (e.g., calcium, vitamin D) and energy deficiency [55]. The diet elimination is usually recommended for 2–4 weeks, followed by symptomatic assessment. In case of no symptoms, CMP can be re-introduced, and the infants should be observed closely for symptomatic relapse. A mother of relapsed infants should continue the diet elimination as long as she is breastfeeding [79].

When exclusive breastfeeding is impossible, a "therapeutic formula" is the first-line management option for infants with mild-to-moderate symptoms. This formula is generally characterized by minimal amounts of CMP, in the form of eHF, or complete deprivation of CMP, such as an AAF [63, 64]. While many factors can govern the choice of suitable therapeutic formula, such as cost, palatability, and availability [82], the eHF is well-tolerated by the majority of formula-fed infants, and it has been advocated as the formula of choice by the European and Middle Eastern guidelines [8, 13]. As mentioned previously, it is rather common in the Middle East to utilize other forms of milk, such as goat's milk, which may exhibit cross-reactivity with cow's milk. Thus, primary care physicians and general pediatricians should be aware of other types of milk or diet with cross-reactivity to cow's milk [13]. On the other hand, the pHF is not recommended by many international guidelines for the treatment of CMPA [55, 83].

AAF is primarily reserved for infants exhibiting reactivity to eHF, nearly 2–10% and 40% of uncomplicated and complicated CMPA, respectively [84]. Intolerance to eHF can lead to persistent symptoms, severe complications, delayed diagnosis, and excessive healthcare expenditure [85]. According to the ESPGHAN guideline, the AAF is indicated in cases with severe symptoms, such as anaphylaxis [8]. While the British Society for Allergy & Clinical Immunology (BSACI) defined the criteria for AAF use as eHF intolerance, severe symptoms, enteropathies, severe non-IgE-mediated diseases, faltering growth, multiple food allergies, and exclusively breastfed infants with allergic symptoms or severe atopic eczema [28].

Alongside eHF and AAF, other formulas are available as alternatives or to fulfill the nutritional requirements of specific subpopulations. For example, a nutritionally complete formula with its fat source being predominantly medium chain triglycerides (MCT) can be used in infants with malabsorptive enteropathy to ensure adequate fat absorption and energy intake [86]. The use of soy-based formula is common in some regions [13]. Soy-based formula is thought to reduce the incidence of allergic disorders compared to other CMP-based formulas [87, 88]. Besides, the cost of soy-based formula is lower than the eHF and, the former is regarded to be more palatable [89]. Recent evidence also has highlighted that soy-based formula did not exhibit adverse effects on the growth and physiological functions of the infants [90]. However, cross-reactivity between soy and CMP has been reported, and nearly 10–15% of the infants aged less than 6 months may be sensitized to soy [13]. In addition, there are concerns regarding the harmful impacts of phytoestrogens content of the soy-based formula on the sexual and endocrine development of the infants, although the current evidence is doubtful [91]. Also, the eHF leads to greater oral tolerance towards CMP than soy-based formula. Thus, the soy-based formula can be considered in infants older than six months in cases of unaffordable eHF or when infants rejected the taste of eHF [92].

Rice-based formula is another CMP-free alternative, with the advantage of affordable cost, good palatability, and nutritional adequacy [93, 94]. However, rice-based formula is available in limited centers within the Middle East, and there is no strong evidence to support the use of rice-based formula, owing to limited data regarding its efficacy and concerns over the arsenic levels [13, 95].

Weaning of infants with confirmed CMPA should be based on a CMP-free diet until a challenge test confirms the tolerance acquisition. However, it is critical to emphasize the importance of not delaying supplementary foods and the gradual introduction of these foods, preferably while the mother is still breastfeeding. Nutritional supervision is crucial during the weaning until CMP-containing food is introduced [55]. New methods of weaning are evolving, such as spoonable yogurt-consistency or semi-solid AAF formula to enhance energy intake, nutritional intake (especially calcium), and tolerability, which was found to be comparable to AAF in children above 6 months of age [96].

Nearly half of the infants with CMPA tolerate CMP by the first year of life; this percentage increases to 75% by the end of the third year of life [89]. Previous guidelines and consensus documents recommended that the eHF should be continued for at least 6 months, alongside nutritional counseling, before re-challenging [55, 82]. However, infants with severe IgE-mediated reactions or high IgE titer usually stay on the eHF for a longer duration (up to 18 months) [8, 29]. A challenge with cow's milk may be performed after maintaining a therapeutic diet for at least three months in a specific IgE negative test or mild symptoms, and at least 12 months in a high-positive IgE test or severe reaction [13]. A recent consensus from the Middle East has proposed a step-down approach in which the pHF-whey (pHF-W) can be used as a bridge between therapeutic formulas and intact CMP in the challenge test. The proposed benefits of this approach include a decrease in the misuse of pHF in the treatment of CMPA and proper management of functional gastrointestinal disorders [10]. Nonetheless, this approach is still not supported by solid clinical evidence, and further trials are required to assess its clinical benefits.

The addition of prebiotics and synbiotics to therapeutic formulas was found to improve tolerance rate at the end of the first year of life [97, 98]. Among infants with non-IgE-mediated reaction, the combination of AAF and synbiotics resulted in improved gut microbiota comparable to that of healthy infants [97].

The statements regarding the management of CMPA that achieved a consensus agreement are provided in Table 2. The panel emphasized the importance of early diagnosis and exclusive or partial breastfeeding. In formula-fed infants, eHF is the first option, while AAF is preserved for infants with red flag signs or reactions to eHF. Therapeutic formula containing MCT is recommended for infants with CMPA and with malabsorptive enteropathy. The CMP re-challenge should be pursued only after a minimum of 6 months for confirmed cases, and this period should be extended to 12–18 months in cases with severe immediate IgE-mediated reactions. The use of AAF with specific synbiotics is recommended.

**Table 2:** Middle East Consensus Statements on the management of CMPA

Statement	Level of agreement (%)
Earlier diagnosis is a factor indicating a good prognosis and may lead to a shorter duration of nutritional management. An earlier diagnosis of CMPA also reduces the cost of CMPA management	93.30
It is recommended that exclusive or partial breastfeeding is continued unless alarm symptoms require different management	93.30
Formulae containing free amino acids as the only nitrogen source are the best option in infants reacting to eHF	93.30
AAF is recommended as a first-line treatment in infants with: infants still reacting on eHF, severe anaphylactic reactions, severe gastrointestinal symptoms, severe eczema, faltering growth, and multiple food allergies	90.90
Partially hydrolyzed formulae based on CMP or other mammalian protein are not recommended for infants with CMPA	100.00
Infants with CMPA and with malabsorptive enteropathy should have formulas with highly reduced allergenicity without lactose and with MCT	100.00
The use of soy-based formulae could be an alternative therapeutic formula. However, there are some concerns regarding the isoflavone (phytoestrogen) content of soy formulae and associated cross-allergy	90.90
If the diagnosis of CMPA is confirmed in infants up to age 12 months, an elimination diet should be maintained using a therapeutic formula for at least 6 months or until 9 to 12 months of age	86.70
Infants/children with severe immediate IgE-mediated reactions should remain on the elimination diet for 12 or even 18 months before they are re-challenged and after repeating specific IgE testing	100.00
A challenge with cow's milk may be performed after maintaining a therapeutic diet for: (1) At least 3 months in specific IgE negative or with mild symptoms; (2) At least 12 months in high-positive IgE test or with a severe reaction	92.90
AAF with specific synbiotics can be considered in children with IgE, non-IgE, or mixed IgE-mediated CMPA	92.90
In exclusively breastfed and formula-fed infants with proven CMPA, weaning food should be free of CMP until a supervised successful oral challenge indicates the development of tolerance	100.00
New methods of weaning are evolving, such as spoon-fed yogurt-type AAF formula to enhance energy intake, nutritional intake (especially calcium), and tolerability, which was found to be comparable to AAF in children above 6 months of age	100.00

*CMPA* cow’s milk protein allergy, *AAF* amino acid formula, *eHF* extensively hydrolyzed formula, *MCT* medium chain triglyceride

### Management algorithm

**Fig. 1:**
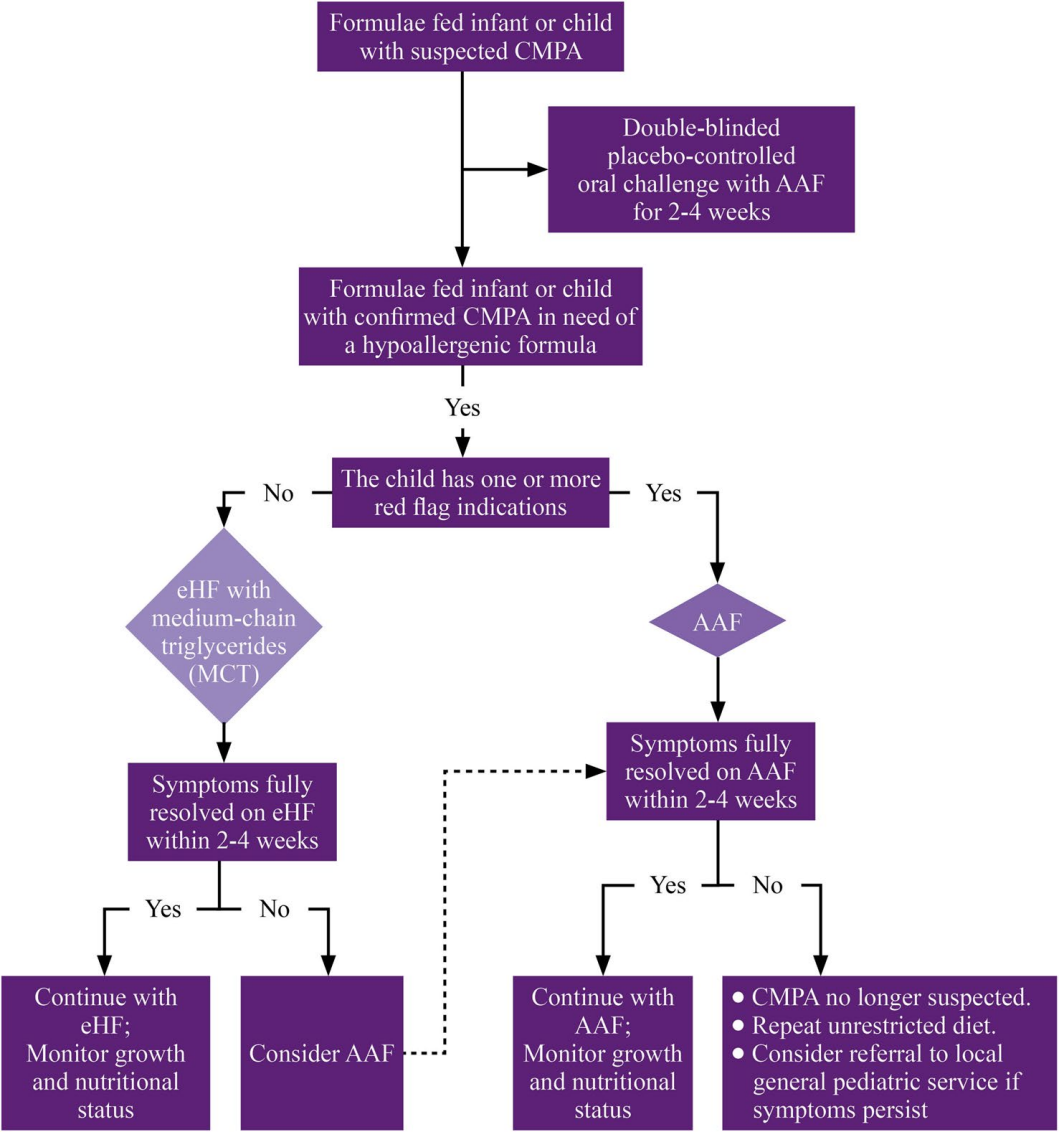
An algorithm for the treatment of CMPA

The panel agreed that in formula-fed infants or children with suspected CMPA, the DBPCFC should be performed for 2–4 weeks using AAF. Formula-fed infants with confirmed CMPA should be offered a therapeutic formula. The eHF with MCT is indicated with no red flag signs. At the same time, the AAF is offered for infants with red flag signs (severe anaphylactic reactions, severe gastrointestinal symptoms, severe eczema, faltering growth, and multiple food allergies). Infants on eHF, who exhibit resolution of symptoms within 2–4 weeks, should continue eHF, with special attention to the growth and nutritional status. On the other hand, AAF should be considered for infants with persistent symptoms. In infants with red flag signs, who are offered AAF, the AAF should be continued if the symptoms resolve within 2–4 weeks, with particular attention to the growth and nutritional status. In contrast, in cases with no symptomatic improvements after AAF, other measures should be followed, including the exclusion of CMPA, a repeat of unrestricted diet, or referral to a specialized center. This algorithm achieved an agreement level of 90.9% (Fig. 1).

### Strengths and limitations

Previously, it was suggested that a minimum of 12 experts was required to ensure the reliability of the Delphi-based consensus [99]. The present survey successfully recruited 15 experts from various Middle East countries, which improved the validity of the final consensus statement. We also utilized a purposive sampling technique to ensure adequate group dynamics during the virtual meeting [100]. The high response rates during each step of the Delphi process was another strength. However, the current process has certain limitations. Although incorporating the virtual meeting in the Delphi process allowed more feedback from the expert and exchange of information, it might have impacted the subject's anonymity and led to the "dominant individuals" effect [14]. Some statements were not supported by level 1 evidence, representing another limitation of the present consensus. The possibility of selection bias during the panel recruitment phase was present as well.

## Conclusions

The estimated prevalence of CMPA in the Middle East ranges from 1 to 5%. The present Delphi-based study combined the best available evidence and clinical experience to optimize the diagnosis and management of CMPA presenting to the healthcare settings in the Middle East. The experts developed several statements and a clinical pathway algorithm to aid primary healthcare physicians and general pediatricians in diagnosis and management of CMPA presenting to primary and advanced healthcare settings in the Middle East. Multidisciplinary collaboration is needed to develop regional consensus regarding the diagnosis and treatment of CMPA in infants and children. The consensus should be comprehensive and should involve all specialties and key players that deal with CMPA to share their ideas and suggestions. Another interesting idea is to develop a national day for CMPA in which experts get together and hence move forward.

## Data Availability

Not applicable.
